# Interrupted time series analysis of free maternity services policy in Nyamira County, Western Kenya

**DOI:** 10.1371/journal.pone.0216158

**Published:** 2019-05-08

**Authors:** Henry Owuor, Asito Stephen Amolo

**Affiliations:** 1 Department of Family Medicine, Moi University, Eldoret Town, Uasin Gishu County, Rift Valley Province, Kenya; 2 Department of Biological Sciences, Jaramogi Oginga Odinga University of Science and Technology, Bondo Town, Siaya County, Nyanza Province, Kenya; Aga Khan University, KENYA

## Abstract

**Introduction:**

The Government of Kenya instituted the free maternity services (FMS) policy to improve utilization of maternal healthcare services and thus improve maternal health. The aim of this study was to evaluate the effect of the FMS policy on the uptake of maternal health services in Nyamira County in western Kenya.

**Methods:**

An interrupted time series study design was used to design the study and to analyze the collected data. Forty-two data sets were collected for each outcome variable i.e. 24 pre- and 18 post-intervention. Monthly data was abstracted from the District Health Information System-2 (DHIS-2) and verified using facility data. The collected data was then keyed into SPSS-17, cleaned and analyzed.

**Results:**

During the study period, there was a significant increase in births attended by skilled attendants up to the 12^th^ month (p<0.05) and caesarean section up to the ninth month (p<0.05). There was a decrease in obstetric complications up to the 12 month (p<0.05). In addition there was a significant increase in institutional maternal mortality ratio (iMMR) in the 12^th^ and 18^th^ months (p<0.05) following the implementation of free maternity service policy. There was a significant increase in deliveries in hospitals from the 1^st^ to the 18^th^ month (p<0.05) whereas in primary health care facilities the increase in deliveries was only significant up to the 6^th^ month (p<0.05).

**Conclusions:**

The FMS policy led to progress towards improving maternal health by improving access to maternal health services. The improved utilization of maternal health services was more marked in hospitals. There was a worsening of institutional maternal mortality ratio.

## Introduction

User fees are charges levied at the point of use of any aspect of health services [1]. User fees for health services were introduced or substantially increased in African countries following the 1987 joint World Health Organization/United Nations Children’s Fund Bamako Initiative whose aim was to address severe problems in the financing of maternity care [1]. User charges for essential drugs were also introduced to generate funds to improve the quality of health services and equity in access to these services [1]. However, user fees have seldom produced these intended benefits [2, 3]. Instead, they have been a significant barrier to access of maternal health care services in Kenya [2]. Studies done between 2003 and 2006 in three African countries (Burkina Faso, Kenya and Tanzania) found that the mean cost of a normal or a complicated delivery in Kenya was US$18.4 compared to US$ 7.9 and US$5.1 for Burkina Faso and Tanzania respectively [4]. This has made user fees a prohibitive barrier to access to essential services even in Kenya [5].

Several countries have experimented with user fee exemptions. Overall, removal of user fees often results in increases in the use of health services [3, 4]. In South Africa, a study found significant increases in care utilization following removal of user fees for pregnant women and children [6]. However this study was carried out in only one facility and its findings may not have been representative of population level changes [6]. A study in Mali showed that user fee exemptions increased uptake for caesarean sections and reduced maternal mortality [7]. Implementation of policies that ban user fees encounter many challenges like health worker dissatisfaction due to lack of incentives, inadequate staffing levels, inadequate supplies e.g. drugs, inadequate information & education activities to the targeted population and a lack of monitoring of the policy implementation process and outcomes [8]. This may alter their effect and result in only short-term effects.

As at 2013 Kenya had made insufficient progress towards improving maternal health and had not achieved its 5^th^ MDG target of at least 147 maternal deaths per 100,000 live births by 2015 since the MMR was 362 maternal deaths per 100,000 live births [9, 10].This high maternal mortality is mainly contributed to by the low utilization of the available maternal healthcare services which among other factors is occasioned by user fees whose re-introduction was part of the health sector reforms driven by international agencies like The World Bank [10–13].

The utilization of maternal health services in Kenya is low [10]. A paltry 61.8% of all deliveries in Kenya and 74.1% of all deliveries in Nyamira County were attended by skilled health personnel [10]. Fifty-eight percent of pregnant women made four or more antenatal care visits during their pregnancy in Kenya whereas in Nyamira County 41% of pregnant women attended four or more ANC visits [10, 14]. The FMS policy was introduced in June 2013. Unlike in the previous user fee abolition policies public health facilities got reimbursement for the lost revenue to deter re-introduction of official or unofficial fees. The reimbursement was based on the level of facility (facility type) and the number of deliveries conducted at the facility. Under this program, primary health care facilities (health centers and dispensaries) are reimbursed Kshs. 2,500 (USD 27.7) for every delivery whereas hospitals are reimbursed KShs. 5,000 (USD 55.6) for every delivery conducted in the facility. Since the inception of FMS in Kenya, no study has been undertaken to assess its effect on the utilization of health facilities for skilled birth attendance.

In this study, we sought to determine the uptake of maternal health services in Nyamira County, Western Kenya eighteen months following the introduction of the free maternity services (FMS) policy in Kenya. Using a methodology proposed in a systematic review by Dzakpasu et al., we use an interrupted time series study design to estimate the effect of the policy on: deliveries attended by skilled health personnel; attendance of fourth ANC; institutional maternal mortality ratio (iMMR); and birth related complications [1]. We will also determine the distribution, by type of health facility, of deliveries attended by skilled birth attendants in the county.

## Materials and methods

This study was approved by Jaramogi Oginga Odinga Teaching and Referral Hospital’s Ethics Review Committee, accreditation number 01713. Since this study involved data abstraction no written consent was obtained from the study participants for using their records. Participant reports/information was already anonymized and de-identified prior to data collection.

### Study design

The Interrupted Time Series (ITS) study design was used to conduct the study. This is a quasi-experimental longitudinal study design which involves statistical comparison of time trends before and after an intervention. It was used since there was a definite point in time when the implementation of the policy began and data could be obtained from the District Health Information System (DHIS-2) for the time period before and after policy implementation. There was no parallel event that would have affected utilization of maternal health services for deliveries supervised by skilled birth attendants in the county. Devolution of health services was delayed and mainly focused on provision of services. However, the study design and the method of analysis would remove the effect of any confounding factors or biases that may affect the results of the study. This is because ITS analysis involves a before-after comparison within a single population, rather than a comparison with a control group. This has the advantage that selection bias and confounding due to between-group differences are limited. The manner in which deliveries from health facilities were captured and reported to the DHIS-2 did not change in any way to affect study findings (i.e. there was no change in mode of measurement). A trend was established for before and after the implementation of the policy (intervention) for each indicator with a goal of determining whether the policy had a significantly greater effect than any underlying secular or seasonal trends.

### Setting

The study was conducted in Nyamira County, one of the forty-seven counties in Kenya. Nyamira County is in the former Nyanza Province and borders Homabay County to the north, Kisii County to the west, Bomet County to the south east and Kericho County to the east. Nyamira County was selected for the study because of its maternal health indicators, particularly uptake of maternal health services and maternal mortality. The County covers an area of 899.4 km^2^ and is home to five sub-counties: Nyamira (South), Nyamira North, Borabu, Manga and Masaba North [15]. The County had a population of 598,252 persons in the 2009 housing and population census report but is projected to have 667,716 persons in 2015 [16].

There are 130 health facilities in the county and eight of these are hospitals. The rest are dispensaries and health centers (i.e. PHCF). The average distance to a health facility in Nyamira County is 7 km and agriculture is the main economic activity with tea and coffee being the main cash crops grown [17].

### Study population and sampling strategy

All the data on the variables of interest (deliveries attended by skilled health personnel; attendance of fourth ANC; institutional maternal mortality ratio (iMMR); and birth related complications) were collected from all the health facilities. These were incorporated in the study so as to achieve population-level outputs & outcomes, and representativeness. Data was therefore collected for all the health facilities in the county.

### Data collection

Data was abstracted from the District Health Information System-2 (DHIS-2) for each variable of interest using a data abstraction form. These were then verified using facility level data. Data was collected for the period between June 1, 2011 and November 30, 2014. Therefore, forty-two data sets were collected for each outcome variable i.e. 24 pre- and 18 post-intervention. A records officer was trained and employed to collect the data from the DHIS-2 and to enter the findings in an excel spreadsheet. Data from hospitals and the PHCF were summated to find the total from hospitals and PHCF respectively. Data was also retrieved by sub-county. The retrieved data was verified by a second officer who counter checked if the entries were similar with what was in the DHIS-2 and with facility level data.

### Data analysis

After data verification and cleaning the data was transferred to the IBM-SPSS version 17 for an interrupted time series (ITS) analysis using the auto-regressive integrated moving-average (ARIMA) model to estimate the intervention effects.

The regression model used for the study given the coefficient β1 is for time, β2 for phase and β3 for interact was:

Variable (e.g. births attended by skilled attendant-SCD) = β0 (constant) + β1time + β2phase + β3interact

Where:

β0 estimates the baseline SCD at the beginning of the pre FMS period
β1 estimates the change in number of SCD that occurs with each month before the FMS policy (pre-slope)
β2 estimates the change in SCD immediately after the FMS policy (interact)
β3 estimates the change in the trend of SCD of the post-FMS period compared to the pre-FMS period (post-slope i.e. β_1_+β_2_)

Estimates for regression coefficients corresponding to two standardized effect sizes are obtained: a change in level (step change i.e. β2) and a change in trend before the intervention (β1). The change in trend after the intervention (β3) is the sum of the pre-intervention slope and the change in level i.e. β1 + β2. Other coefficients generated from the ‘ARIMA model Parameters’ including the corresponding standard error (SE) and the t-values were important in calculating the 95% confidence intervals. The p-values demonstrated the significance of the effect of the free maternity services policy. Percentages were calculated to estimate the relative effect of the policy. This facilitates comparison between the sub-counties and health facilities. The analysis was done to determine the first- and third-month level effect after the policy execution date to monitor any immediate and short-term effects of the policy. Analysis for the sixth and ninth months was to demonstrate any mid-term effects whereas the analyses done at the twelfth and eighteenth month post intervention was to assess whether the policy had a long-term effect. Statistical significance was set at a p≤0.05.

## Results

Table 1 depicts the population and service data before and after the FMS policy implementation. The populations shown represent the mid-period populations before and after the policy’s implementation. There was a 3.7% increase in the population and a 50.9% increase in deliveries attended by skilled health personnel. This was accompanied by increases in fourth ANC clinic visits and average number of CSs per month and a decline in the number of birth-related complications per month. However, there were no major differences in institutional maternal and neonatal deaths. There was an increase in deliveries attended by skilled personnel in all the sub-counties except in Borabu sub-county.

**Table 1:** Population and service data before and after the free maternity services (FMS) policy for Nyamira County.

	Pre-intervention	Post-intervention
**Population data**	**June 2011-May 2013**	**June 2013-Nov 2014**
County Pop	637,295	661,106
Expected Deliveries	21,668	22,478
Average expected deliveries per month	902.8	1248.8
Number of Hospitals	8	8
Number of PHCF	114	122
Total No of Health Facilities	122	130
Number of Sub-County	5	5
**Service Data**		
Hospital Deliveries	7,972	8,667
PHCF Deliveries	12,868	14,903
Total Facility based Deliveries (ratio)	20,840(96.2%)	23,570(104.9%)
Average facility deliveries per month	868.3	1309.4
Fourth ANC visits and (ratio)	14,947 (69.0)	14,051 (62.5%)
Average 4^th^ ANC per month	622.8	780.6
Caesarean Sections done and (ratio)	753(3.6%)	809(3.4%)
Average CS per month	31.4	44.9
Maternal Deaths	21	18
Complications	84	22
Neonatal Deaths	141	142
**SCD by Sub-Counties**		
Borabu	3,250	3,224
Manga	1,986	2,171
Masaba North	3,775	4,813
Nyamira South	9,148	9,326
Nyamira North	3,762	4,886

**Fig 1:**
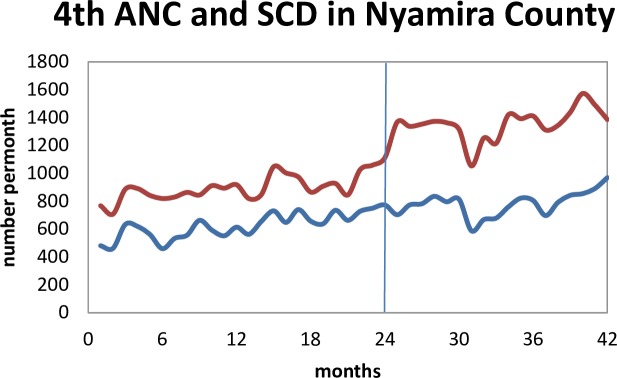
Trends for SCD (red/upper curve) and 4^th^ ANC (blue/lower curve) before and after implementation of the free maternity services policy (the line at 24 months).

Fig 1 shows the trend of the total monthly deliveries attended by skilled health personnel and the total number of pregnant women attending a fourth antenatal care clinic in the county. There was a change in level and trend after the policy implementation.

There was an immediate increase in the number of deliveries attended by skilled birth attendants while there was no effect in the 4^th^ ANC trend following FMS policy implementation.

Table 2 shows the gradients, the level effects and the relative effects of the policy by month for SCD and fourth ANC attendance. There had been an increase of 10 SCD per month before the implementation of the FMS (95% CI: 3.4–16.8; p = 0.004). Following the implementation of the policy there was an immediate increase of 257 deliveries attended by skilled health personnel (p<0.001). 24% of this increase is directly attributed to the policy. There was a long-term effect of the policy since 228 (18% of which was due to the policy) more deliveries occurred in the 18^th^ month (-4.9–461.2, p = 0.055). The increase in deliveries attended by skilled health personnel was statistically significant from the 1^st^ to the 12^th^ month.

**Table 2:** Parameter estimates for SCD and 4th ANC from the ARIMA model for the FMS policy (June 2011 to Nov 2014).

	Co-efficient	SE	t-statistic	p-value	95% CI	RE %
**I) SCD**						
Fit Test	Stationary R^2^ = 0.904; Ljung-Box Q(18) = 19.938 (p = 0.277; d.f. = 17)					
Pre-Slope (β_1_)	10.086	3.305	3.051	0.004	3.4–16.8	-
Interact (β_2_)	-1.895	5.982	-0.317	0.753	-14.0–10.2	-
Post-Slope (β_1_+ β_2_)	8.191					
**Level effect**						
1	257.4	66.047	3.898	<0.001	123.6–391.3	24.4
3	254.0	65.197	3.896	<0.001	121.9–386.1	25.1
6	248.8	68.270	3.011	0.001	110.5–387.1	22.8
9	243.6	75.919	3.209	0.003	89.8–397.5	22.6
12	238.5	86.945	2.743	0.009	62.3–414.6	20.9
18	228.1	115.02	1.983	0.055	-4.9–461.2	18.7
**II) 4ANC**						
Fit Test	Stationary R^2^ = 0.731; Ljung-Box Q(18) = 15.261 (p = 0.577; d.f. = 17)					
Pre-slope	10.720	2.571	4.169	<0.000	5.5–15.9	-
Interact	-1.233	4.853	-0.254	0.801	-11.1–8.6	-
Post-Slope	8.487					
**Level Effect**						
1	-57.0	51.597	-1.105	0.276	-161.5–47.5	-7.4
3	-53.4	49.684	-1.074	0.290	-154.0–47.3	-6.7
6	-63.2	52.766	-1.197	0.239	-170.1–43.7	-7.6
9	-66.9	58.503	-1.143	0.260	-185.4–51.6	-8.2
12	-70.6	66.968	-1.054	0.299	-206.3–65.1	-8.0
18	-78.0	88.772	-0.878	0.385	-257.8–101.9	-8.2

There had been 11 more women attending the fourth ANC in the county per month (95% CI: 5.5–15.9; p<0.001) before the implementation of the FMS policy (Table 2). The overall effect of the policy on attendance of the fourth ANC was negative and ranged between 53 to 78 fewer fourth ANC attendances. However this was not statistically significant.

The number of deliveries attended by skilled birth attendants and caesarean sections conducted at the county referral hospital had been static (Table 3). However, there were 12 more caesarean sections per month immediately following policy implementation (95% CI: 0.4–24.3; p = 0.043). 40% of these CSs are directly attributed to the FMS policy. The increase in the number of CSs was statistically significant up to the 9th month and was therefore medium -term.

**Table 3:** Parameter estimates for SCD, CS and iMMR at the CRH using ARIMA model for the FMS policy (June 2011 to Nov 2014).

	Co-efficient	SE	t-statistic	p-value	95% CI	R.E. (%)
**I) SCD at the CRH**						
Fit Test	Stationary R^2^ = 0.486; Ljung-Box Q(18) = 16.501 (p = 0.489; d.f. = 17)					
Pre-slope	-0.466	0.797	-0.584	0.563	-2.1–1.1	-
Interact	2.265	1.467	1.544	0.131	-0.7–5.2	-
Post-slope	1.799					
**Level Effect**						
1	33.679	16.595	2.029	0.050	0.1–67.3	23.8
3	37.449	16.083	2.328	0.025	4.9–70.0	26.9
6	45.006	16.798	2.679	0.011	11.0–79.0	32.4
9	51.802	18.382	2.818	0.008	14.6–89.0	38.3
12	58.598	20.793	2.818	0.008	16.5–100.7	42.5
18	72.198	27.155	2.658	0.012	17.2–127.2	55.6
**II) CS at the CRH**						
Fit Test	Stationary R^2^ = 0.337; Ljung-Box Q(18) = 16.549 (p = 0.485; d.f. = 17)					
Pre-Slope	0.003	0.279	0.010	0.992	-0.6–0.6	-
Interact	0.159	0.511	0.311	0.757	-0.9–1.2	-
Post-Slope	0.162					
**Level Effect**						
1	12.330	5.887	2.094	0.043	0.4–24.3	40.2
3	12.204	5.721	2.133	0.040	0.6–23.8	38.2
6	13.125	5.921	2.217	0.033	1.1–25.1	40.7
9	13.602	6.447	2.110	0.042	0.5–26.7	45.6
12	14.079	7.264	1.938	0.060	-0.6–28.8	47.4
18	15.033	9.449	1.591	0.120	-4.1–34.2	46.1
**III) iMMR at the CRH**						
Fit-Test	Stationary R^2^ = 0.365; Ljung-Box Q(18) = 7.574 (p = 0.975; d.f. = 17)					
Pre-Slope	-16.800	6.474	-2.595	0.013	-30.0 to -3.7	-
Interact	27.065	12.110	2.235	0.032	2.5–51.6	-
Post-Slope	10.265					
**Level Effect**						
1	84.452	127.816	0.661	0.513	-174.5–343.4	-
3	126.011	123.220	1.023	0.313	-123.6–375.7	-
6	219.779	132.098	1.664	0.105	-47.9–487.4	-
9	300.976	147.097	2.046	0.408	3.0–599.0	-
12	382.173	168.714	2.265	0.029	40.4–724.0	-
18	544.568	223.691	2.434	0.020	91.4–997.8	-

In contrast, there was a long-term effect of the of the FMS policy on deliveries supervised by skilled birth attendants in the same facility. There were 34 more deliveries immediately following policy implementation (95% CI: 0.1–67.3; p = 0.050) and 24% of these were due to the policy. The relative effect of the policy on births attended by skilled health personnel was statistically significant up to the 18^th^ month.

Before the FMS policy implementation, there had been 17 fewer maternal deaths per 100,000 live births per month (95% CI: -30.0 to -3.7; p = 0.013) at the county referral hospital (Table 3). After the policy there was a steady increase in iMMR for the hospital. This increase was not statistically significant until the ninth month after which the 301 more maternal deaths per 100,000 live births per month was statistically significant (95% CI: 3.0–599.0; p = 0.048). The iMMR for the 12^th^ and 18^th^ month were also statistically significant.

There had been no change in the trend of obstetric complications in the hospital per month before the policy implementation (see Table 4). Following the implementation of the FMS policy, there were 3 fewer cases of obstetric complications per month which was statistically significant up to the 12^th^ month. Table 4 also demonstrates that there was no significant effect of the policy on neonatal mortality ratio after the FMS policy implementation.

**Table 4:** Parameter estimates for obstetric complications and neonatal mortality ratio in CRH using the ARIMA model for the FMS policy (June 2011 to Nov 2014).

	**Co-efficient**	**SE**	**t-statistic**	**p-value**	**95% CI**	**RE %**
**I) O.C. (PPH, Obstructed Labor)**						
Fit Test	Stationary R^2^ = 0.269; Ljung-Box Q(18) = 19.203 (p = 0.317; d.f. = 17)					
Pre-Slope	0.046	0.054	0.848	0.402	-0.1–0.2	-
Interact	-0.026	0.099	-0.264	0.793	-0.2–0.2	-
Post-Slope	0.020					
**Level effect**						
1	-3.043	1.151	-2.644	0.012	-5.4 to -0.7	-
3	-3.087	1.115	-2.768	0.009	-5.3 to -0.8	-
6	-3.174	1.155	-2.591	0.014	-5.5 to -0.8	-
9	-3.253	1.255	-2.591	0.014	-5.8 to -0.7	-
12	-3.331	1.413	-2.358	0.024	-6.2 to -0.5	-
18	-3.489	1.835	-1.901	0.065	-7.2 to 0.2	-
**II) Neonatal Mortality Ratio**						
Fit Test	Stationary R^2^ = 0.046; Ljung-Box Q(18) = 12.300 (p = 0.782; d.f. = 17)					
Pre-slope	0.080	0.138	0.580	0.565	-0.2–0.4	-
Interact	-0.105	0.252	-0.416	0.680	-0.6–0.4	-
Post-Slope	-0.025					
**Level Effect**						
1	-2.327	2.911	-0.799	0.429	-8.2 to 3.6	-
3	-2.545	2.834	-0.898	0.375	-8.3 to 3.2	-
6	-2.850	2.954	-0.965	0.341	-8.8 to 3.1	-
9	-3.164	3.225	-0.981	0.333	-9.7 to 3.4	-
12	-3.478	3.636	-0.957	0.345	-10.8 to 3.9	-
18	-4.106	4.720	-0.870	0.390	-13.7 to 5.5	-

Table 5 shows the analysis of deliveries attended by skilled health personnel in hospitals and PHCF. There had been no change in the number of deliveries attended by skilled health personnel per month prior to the implementation of the policy in the hospitals (95% CI: -4.1–3.3; p = 0.833) whereas there had been eight more deliveries per month at the PHCF (95% CI: 3.6–14.3; p = 0.002). Immediately following the policy implementation, there were 116 more deliveries in the hospitals per month (95% CI: 39.0–193.0; p = 0.004). 34% were directly due to the policy. There was a long-term effect of the policy on SCD in hospitals (up until the 18^th^ month). However the effect of the policy on SCD in PHCF was short-term (up to the 6^th^ month).

**Table 5:** Parameter estimates for the SCD by Hospitals and PHCF using the ARIMA model for FMS policy (June 2011 to Nov 2014).

	Co-efficient	SE	t-statistic	p-value	95% CI	RE %
**I) Hospitals**						
Fit Test	Stationary R^2^ = 0.700; Ljung-Box Q(18) = 11.989 (p = 0.801; d.f. = 17)					
Pre-Slope	-0.390	1.833	-0.213	0.833	-4.1–3.3	-
Interact	5.458	3.373	1.618	0.114	-1.4–12.3	-
Post-Slope	5.068					
**Level effect**						
1	115.989	38.009	3.052	0.004	39.0–193.0	34.27
3	126.538	36.786	3.440	0.001	52.0–201.1	38.15
6	143.280	38.582	3.714	0.001	65.1–221.4	42.46
9	159.656	42.283	3.776	0.001	74.0–245.3	49.20
12	176.031	47.874	3.677	0.001	79.0–273.0	53.12
18	208.781	62.565	3.337	0.002	82.0–335.4	63.34
**II) Primary Health Care Facilities (PHCF)**						
Fit Test	Stationary R^2^ = 0.863; Ljung-Box Q(18) = 14.355 (p = 0.642; d.f. = 17)					
Pre-slope	8.967	2.644	3.391	0.002	3.6–14.3	-
Interact	-9.224	4.948	-1.864	0.070	-19.2–0.8	-
Post-Slope	-0.257					
**Level Effect**						
1	189.427	52.300	3.622	0.001	83.4–295.4	28.72
3	184.745	51.680	3.580	0.001	80.2–289.3	28.46
6	143.303	53.985	2.655	0.012	33.9–252.7	20.36
9	115.629	60.078	1.925	0.062	-6.0–237.3	16.86
12	87.955	68.884	1.277	0.210	-51.6–227.5	12.03
18	32.605	91.314	0.357	0.723	-152.4–217.6	4.00

## Discussion

### Key findings

Following the implementation of the policy there was an immediate and significant increase in deliveries attended by skilled health personnel at the county level. This increase in SCD was long-term as it remained significant in the 18^th^ month. This was however associated with a decline in attendance of fourth ANC which was not statistically significant. At the CRH, there was a long-term increase in SCD and a medium-term increase in the number of CSs. At the same facility, there had been a significant decline in iMMR. However, there was an increase in iMMR after the policy that became significant from the 9^th^ month onwards. There was a statistically significant medium-term (up to one year) reduction in number of obstetric complications. However, there was no significant effect of the policy on neonatal mortality. In addition, there was a significant long-term effect of the policy on SCD in hospitals (up until the 18^th^ month). However the effect of the policy on SCD in PHCF was short-term (up to the 6^th^ month).

### Discussion of key findings

The FMS policy improved the utilization of maternal health services, particularly the deliveries attended by skilled birth attendants. The relative effect of the policy was more marked in the hospitals than in PHCF. This improvement in deliveries attended by skilled personnel in the county was expected as studies have shown that facility deliveries usually increase following similar fee exemption policies [18–20]. The larger improvement of deliveries in hospitals was not expected though since the PHCF are most accessible to the populace [21]. The initial increase in utilization of maternity services at the PHCF may be due to their proximity and comparative ease of accessibility [21, 22]. Many pregnant mothers in labor would have been reluctant to seek care at the hospitals because of distance. There could also have been a misconception that the free services were only available at the PHCF and not at the hospitals.

Later on, due to quality of care (occasioned by staff numbers and qualifications) and the 24 hour availability of staff at the hospitals, there was a shift of clients from PHCF to hospitals resulting in the sustained rise in number of deliveries attended by skilled health personnel in the hospitals [23]. In addition, the hospitals received a relatively larger reimbursement per delivery compared to the PHCF [24]. Hospitals could therefore offer better quality services, better staff rewards and better gift hampers to mothers that deliver at their facilities.

Previous studies have shown that facility deliveries usually increase following similar fee exemption policies [18–20]. This is despite methodological limitations including a lack of secular time trends (temporal trends) and population representativeness of the sample for these studies [1]. Nonetheless, a study that estimated effects net of temporal trends reported an increase of 7.3 monthly facility deliveries in district hospitals in Afghanistan immediately following fee removal [8]. This was not statistically significant and the initial increase was followed by a slight decline in trend [8]. The improvement in deliveries conducted by skilled attendants is higher in our study because in Afghanistan 83.6% of deliveries were free before the ban. Similar to our findings this study also reported more marked increase in deliveries attended by skilled attendants in the hospitals [8].

Cost had been a significant barrier to maternal health care services in Kenya. Studies done in 2003 and 2006 in three African countries (Burkina Faso, Kenya and Tanzania) reported that the mean cost of a normal or a complicated delivery was US$18.4 compared to US$ 7.9 and US$5.1 for Burkina Faso and Tanzania respectively [4]. The mean out-of-pocket expenses for normal delivery care at Kenyan health centers and dispensaries was US$7.4 and US$4.3 respectively whereas it was US$ 13.5 in government hospitals with costs at private/mission facilities being twice as high as those incurred at government facilities [4]. The average cost of a complicated delivery (defined as either a delivery complicated by hemorrhage, eclampsia or obstructed labor) was US$26.0 and US$68.7 at government hospitals and private facilities respectively, which were very high compared to US$6.7 and US$8.0 incurred at health centers and dispensaries respectively [4]. Therefore the FMS policy eliminated a significant barrier to access in a county where many people are poor as attested to by the human development indices in the county which are actually lower than the national indices [15]. This explains the long-term increases in SCD.

Another effect of the policy is the sustained decrease in delivery-related maternal complications (i.e. post-partum hemorrhage and obstructed labor). Better labor management from the available staff contributed to the long-term decline in obstetric complications at the facility. However, there was no effect on neo-natal mortality ratio. This differs from findings in other studies conducted in the region. In Ghana, Senegal and Nepal there was an increase in the number of pregnant women presenting with pregnancy- and delivery-related complications including hypertension, hemorrhage and obstructed labor. This was attributed to increased access to emergency obstetric services [25–27]. These studies however did not take into account any underlying trends.

Increased referral of emergency cases requiring surgical intervention from PHCF could have resulted in the short-term increase in the number of caesarean sections (CSs) conducted at the CRH. This later waned in significance when there was a shift of clients from the PHCF to the hospital. These patients had their labor managed better than in the PHCF hence the stalled effect of the policy on CSs. In Senegal, a fee ban on caesarean deliveries resulted in the increase of caesarean deliveries from 4.2% to 5.6% over one year. This was a cross-sectional study that failed to take care of any underlying trends [27]. Meanwhile, an increase in caesarean deliveries from 0.9% to 2.3% was reported in Mali following fee ban on caesarean sections in 2009 [5]. There was limited access to caesarean deliveries to a section of the population (the poor) in Mali since there was no fee ban on normal deliveries.

Lack of a maternity theatre and fatigue due to high workload could have caused delays in performing emergency CSs. There could have been deterioration in quality of maternity services due to staff shortage. In addition, the obstetrician at the CRH resigned. This explains the worsening of the facility’s iMMR which had been declining before the policy implementation. The rising iMMR is in agreement with a study done at tertiary hospitals in South Africa that reported an increasing iMMR following a fee ban. The authors also postulated that this was due to a decline in quality of maternal health services resulting from an increase in patient load with no increase in staff or facility resources [28]. However, the study did not estimate the effect attributable to the fee change. These findings do not agree with similar studies in Sub-Saharan Africa that reported a decline in iMMR following fee exemptions. In Ghana there was a non-significant decline from 34% to 10% [26]. Findings in Mali reported a decline in post-caesarean maternal deaths that was attributed to early seeking of emergency care following the fee ban [5].

Contrary to the increase in facility utilization for deliveries seen since the introduction of the free maternity services policy, there was a trend towards lower attendance for the fourth ANC clinic. ANC services had been free in the country [29]. This is comparable to the findings of a similar study in Afghanistan where there was an increase in ANC services which had been free before the ban (8). Moreover, user fees on curative services had also been banned in PHCF (dispensaries and health centers). The resultant congestion together with the reduction in consultation time due to the increased workload caused the reductions in 4^th^ ANC by discouraging some women from attending the required number of ANC visits [6].

This study’s main strength is that deliveries attended by skilled personnel in all the facilities in the study area were summated to achieve population representativeness. Others include the quality and quantity of data that was collected to draw the time series. The interrupted time series study design and analysis method eliminated the effect of the trends before policy implementation. There was no other intervention during the study period that could interfere or confound the effect of the policy. The study analyzed the effect of the policy on a multiplicity of health indicators so as to assess the complex nature of real-world environments. It encompassed 24 months pre-intervention and 18 months post-intervention with the longer pre intervention period vital for modeling any cyclical trends hence limiting errors in analysis. The post-intervention period allowed assessment of short-term and long-term effects of the policy.

The study’s limitations include the relatively shorter period and lack of a comparison group since the policy was implemented nationally. This is mitigated by the quasi-experimental study design (interrupted time series) that compares the trends before and after the study i.e. there is still a comparator. This study was in a rural county and its findings may therefore not be generalizable to urban areas and towns. Most of the country remains rural though. The roll out of the policy occurred independent of other factors that could have affected maternal mortality or that could have increased access to skilled health attendance at birth but given its ecological nature other factors that may contribute to the observed change may not be easily identified.

## Conclusion

Since the start of the nationwide implementation of the free maternity services policy in June 2013, there has been progress towards improving maternal health, especially access to maternal health services, in Kenya. The improved utilization of maternal health services was more marked in hospitals. However, there is a worsening of iMMR and an insignificant decline in ANC attendance. Longer-term studies should be conducted to further characterize the effect of the policy. In addition, qualitative studies should be carried out to understand the barriers to hospital delivery in Nyamira County in the context of the FMC policy. Meanwhile PHCF should leverage on their accessibility to improve utilization of health services in these facilities.

## Supporting information

S1 File (XLSX)

## Abbreviations

FMS: free maternity services
iMMR: institutional maternal mortality ratio
DHIS-2: District Health Information System-2
MDG: Millennium Development Goals
US$: United States dollar
MMR: maternal mortality rate
KDHS: Kenya Demographic Health Survey
ANC: antenatal clinic
ITS: Interrupted Time Series
PHCF: primary health care facilities i.e. dispensaries and health centers
CIDP: County Investment and Development Plan
ARIMA: auto-regressive integrated moving-average
SCD: skilled care deliveries, i.e. deliveries attended by skilled health personnel
CSs: caesarean sections
CRH: County Referral Hospital
